# Phenotypic Screening Identifies Synergistically Acting Natural Product Enhancing the Performance of Biomaterial Based Wound Healing

**DOI:** 10.3389/fphar.2017.00433

**Published:** 2017-07-18

**Authors:** Srinivasan Sivasubramanian, Gayathri Chandrasekar, Sara Svensson Akusjärvi, Ramar Thangam, Malairaj Sathuvan, R. B. S. Kumar, Hawraa Hussein, Savariar Vincent, Balaraman Madhan, Palani Gunasekaran, Satish S. Kitambi

**Affiliations:** ^1^Department of Virology, King Institute of Preventive Medicine and Research Chennai, India; ^2^Department of Microbiology, Tumor and Cell Biology, Karolinska Institutet Solna, Sweden; ^3^Council of Scientific and Industrial Research – Central Leather Research Institute Chennai, India; ^4^Center for Environmental Research and Development, Loyola Institute of Frontier Energy, Loyola College Chennai, India

**Keywords:** phenotypic screening, PDD, wound, amnion, biomaterials, biomarkers, natural products

## Abstract

The potential of multifunctional wound heal biomaterial relies on the optimal content of therapeutic constituents as well as the desirable physical, chemical, and biological properties to accelerate the healing process. Formulating biomaterials such as amnion or collagen based scaffolds with natural products offer an affordable strategy to develop dressing material with high efficiency in healing wounds. Using image based phenotyping and quantification, we screened natural product derived bioactive compounds for modulators of types I and III collagen production from human foreskin derived fibroblast cells. The identified hit was then formulated with amnion to develop a biomaterial, and its biophysical properties, *in vitro* and *in vivo* effects were characterized. In addition, we performed functional profiling analyses by PCR array to understand the effect of individual components of these materials on various genes such as inflammatory mediators including chemokines and cytokines, growth factors, fibroblast stimulating markers for collagen secretion, matrix metalloproteinases, etc., associated with wound healing. FACS based cell cycle analyses were carried out to evaluate the potential of biomaterials for induction of proliferation of fibroblasts. Western blot analyses was done to examine the effect of biomaterial on collagen synthesis by cells and compared to cells grown in the presence of growth factors. This work demonstrated an uncomplicated way of identifying components that synergistically promote healing. Besides, we demonstrated that modulating local wound environment using biomaterials with bioactive compounds could enhance healing. This study finds that the developed biomaterials offer immense scope for healing wounds by means of their skin regenerative features such as anti-inflammatory, fibroblast stimulation for collagen secretion as well as inhibition of enzymes and markers impeding the healing, hydrodynamic properties complemented with other features including non-toxicity, biocompatibility, and safety.

## Introduction

Amnion or its major component, collagen proteins, offer an affordable source to develop biomaterials for various therapeutic purposes as the non-immunogenic membrane represents an advantageous source of progenitor cells, tissue regenerative growth factors and substances, and various types of collagen (Ilic et al., 2016). The ultra-structure of these biomaterials indicates that they can be also used as a carrier of small molecules, drugs or in cell therapy in order to augment their healing capability (Das and Baker, 2016; Rahimnejad et al., 2017). Bio-actives and extracts from natural products offer an excellent opportunity to compliment biomaterial preparation in order to reinforce its potential while maintaining affordability. Biomaterial based wound healing approach offers an excellent opportunity to modulate local wound environment so as to produce systemic effects that promote wound healing. Both local wound environment and individual’s physiological status contribute toward wound healing. Local wound environment in chronic conditions is characterized by the presence of excess exudates with elevated protease activity, defective extracellular matrix (ECM) and failure of epithelialization and vascularisation that produce systemic effects on the physiology (Harding et al., 2002). These effects stimulate the production of chemokines resulting in elevated levels of inflammatory cells in the wound environment to delay healing. In addition, other clinical impediments such as diabetes, hypertension, other ailments, infections and patient’s health status are the factors that contribute to delay in healing (Harding et al., 2002). These studies indicate that both local wound environment and systemic effects should be sought in case of impaired healing (Reinar et al., 2008). Testament to that is the report where enhanced biomaterial based tissue regeneration is seen upon modulation of the adaptive immunity (Sadtler et al., 2016). Biomaterials offer a way to orchestrate gradual change at wound site (Salamone et al., 2016) and hence have been tried as wound dressing for delivery of drugs, growth factors or cell therapy with varying degree of success (Das and Baker, 2016; Rahimnejad et al., 2017). We had earlier demonstrated an uncomplicated way to modulate local wound environment using amnion derived biomaterial to heal chronic wounds that were unresponsive to other treatments in leprosy-cured individuals (Srinivasan et al., 2017, under review). This was based on a rationale similar to physiological acclimation to chemical, biological, and environmental stressors, as acclimatization to chronic wound might also be a reason inhibiting healing. Therefore, instead of direct therapeutic intervention, biomaterial based gradual alteration to wound site allows individuals physiology to participate in healing.

In this study, we report a phenotypic screening using bioactive natural products and quantify collagen types I and III from human foreskin derived fibroblast cells. Post screening, we develop a biomaterial with it and characterize its biophysical properties, *in vitro* and *in vivo* effects. In addition, we demonstrate that using this approach we could harness salient features associated with each component. Moreover, we use this material to stabilize wound structure, perform gradual alteration to wound site so as to absorb exudates and get digested, keep the wound moist and aerated, decrease microbial load so as to promote healing.

## Materials and Methods

### Standard Care and Ethical Permits

All animal works were performed in accordance with the national guidelines and local ethical committee constituted at Loyola College, Chennai, CSIR-Central Leather Research Institute, Chennai or Karolinska Institutet, Sweden. Wild-type AB strain zebrafish were housed under standard conditions of day night cycle, feeding and care, and egg and embryos were obtained via natural mating and staged according to Kimmel et al. (1995) Zebrafish embryos were staged in hours or days post fertilization (hpf or dpf), anesthetized using 0.1% Tricaine, kept on ice and fixed using 4% paraformaldehyde (PFA) overnight, and washed with phosphate buffered saline (PBS) containing 0.1% Tween-20 (PBSTw). Wild-type swiss albino mice of both sexes were used in the study. Mice were individually and spaciously housed and experiments were performed according to approved protocols with constant monitoring by in-house veterinarian. Mice were anesthetized with isoflurane, perfused with PBS followed by 4% PFA as previously described (Deferrari et al., 2003; Phiel et al., 2003). Skin samples were dissected out of the perfused mice and transferred into 4% PFA in PBS overnight at 4°C.

### Cell Culture

Human foreskin derived fibroblasts were cultured in DMEM supplemented with 10% fetal bovine serum (FBS) and 1X penicillin-streptomycin (Pen-Strep) (all from Invitrogen). Confluent cells were split 1:3 to 1:5 using TrypLE Express (Invitrogen). Cells were dissociated with trypsinization (Tryple E^TM^ Express 1x, Gibco).

### Phenotypic Screening

Natural product based phenotypic screening was conducted using 120 compounds obtained from Sigma or from NCI Natural Product Set III obtained from National Cancer Institute, United States. The screening was conducted using a final concentration of 5 μM of the compound.

Human fibroblast cells were as above and dissociated, suspended into culture medium without serum and re-plated in 384 well plate (Corning) to an amount of 1000 cells in 50 μl per well. The cells were allowed to settle for 12 h following which the media was replaced with 45 μl of fresh media without serum. After acclimatization for 1 h, 5 μl of compound to a final concentration of 5 μM was added to each well. The plates were then incubated for 72 h at 37°C in cell culture incubator. Post incubation, the media was removed and cells were fixed using 4% PFA for 20 min at room temperature (RT) and washed twice with PBS, 5 min each wash. Post washing, cells were stained with Picro Sirius Red Staining Kit according to manufacturer’s instruction. In brief, cell were stained with Picro Sirius Red for 60 min at RT followed by washing twice with acetic acid and once with 100% ethanol, all supplied in the kit. Following wash, the cells were counter stained with HOECHST 33342 (Invitrogen) and 50 μl PBS was added to each well and the plate was taken for imaging. Imaging was done using Operetta^TM^ High Content Screening System (Perkin Elmer) using Harmony 3.5 software as described before (Kitambi et al., 2014). Imaging was done using excitation and emission spectra of Rhodamine (ex. 538–562 nm, em. 570–640 nm) to visualize Type I collagen and FITC (ex. 450–490 nm, em. 500–550 nm) to visualize Type III collagen, HOECHST and bright field. Nine images were obtained from each well and processed using image processing script (Sazabo et al., 2017) to generate composites which were then taken for image analysis. Image analysis was done using ImageJ, total number of nuclei was counted for each composite image in addition to the total fluorescent intensity. All images producing equal intensity to control cells were filtered out. The compounds producing an increase or decrease/lethal of intensity was then used to generate values showing average intensity per cell and plotted as heat maps using Microsoft excel and Images were processed using Photoshop software for publication.

### Biomaterial Preparation

To prepare KC material, red seaweed *Kappaphycus alvarezii* was collected in the coastal areas of Gulf of Mannar-Mandapam south east coast of India in December 2015. Thallus was cleaned manually with sea water to eliminate epiphytes and then sun-dried at ambient temperature before to be stored in aerated bags in a shaded and ventilate site. Before polysaccharide extraction, the sample was washed abundantly with water and dried for 30 h at 65°C. Carrageenan extraction was carried out according to the previously described procedure (Yermak et al., 2006). In brief, dried algae (2 g) were suspended in 0.5% KOH (100 ml) and 90°C for 3 h in a boiling water bath. The suspensions were centrifuged (10,000 rpm, 20 min, 30°C) and the algal residues were re-extracted twice 2 h in a boiling water bath. The supernatants were pooled and concentrated under vacuum to about 100 ml. The polysaccharides were precipitated with 10% KCL according to previously published procedure (Volod’ko et al., 2016). The gel were washed against distilled water, lyophilized, and then used without further treatment. The total sugar content was determined using the phenol-sulfuric acid method and the sulfate content was measured according to Dodgson and Price (1962). Total protein was determined by the method of Bradford (1976). Uronic acid was determined by carbazole method (Bitter and Muir, 1962). The 1 g of lyophilized *kappa-carrageenan* material was then dissolved in 50 ml distilled sterilized water (pH 7.0) by constant stirring to generate KC solution. Post stirring, KC solution was poured onto a sterile steel tray and incubated at 70°C incubator overnight to generate KC membrane. KC membranes were then taken as sheets or cut into 1 square centimeter patches. To generate Amn-KC membrane, 1 g of lyophilized Amn material was sonicated in KC solution to generate Amn-KC solution. Post sonication, the Amn-KC solution was processed similarly as KC solution to generate sheets or patches.

### Biomaterial Sterilization

Biomaterials were washed extensively with 0.1 M PBS containing antimicrobial chemicals such as 25–50 μM each of amphotericin B, gentamycin, chlorhexidine and metronidazole and immersed in the said antimicrobial solution for 30 min. Post chemical disinfection, the membrane was spread on to the filter paper and kept in the Biosafety Level II Laminar hood and subjected to UV sterilization for 30 min. Post UV sterilization, the material was radiosterilized using 25 kGy ^60^Co gamma radiation.

### 3D Scaffold Preparation and Cell Growth

KC was incubated in DMEM with 10% (FBS) and 1X Pen-Strep for 2 days and allowed to absorb medium and swell. The swollen membrane was manually crushed in a 1.5 ml microcentrifuge tube to generate a paste. Human foreskin fibroblast cells were trypsinized from a cell culture plate and collected as cell pellet in a 2 ml microcentrifuge tube to which the KC paste was added and mixed to generate an uniform dispersion of cells in KC. The paste was then squeezed as rectangular blocks inside a 6 cm petri dish and incubated in a humidified chamber for 2 days. Following incubation, the blocks were transferred to 10 ml of 4% PFA solution and incubated for 30 min at RT. Post fixation, the blocks were transferred to 10 ml of PBS containing DAPI and incubated for 10 min and washed twice with PBS solution (10 min for each wash) and photographed in bright field and DAPI using fluorescent microscope. Images were processed in Adobe Photoshop software.

### Atomic Force Microscopy

Atomic Force Microscopy (AFM) on biomaterials was performed to assess the surface structure of biomaterials. Biomaterials were assessed in tapping mode with Bruker FastScan system using FastScan A cantilever (ω_o_ = 1.6 MHz). Height images were collected in 3D, and amplitude and phase images were collected in 2D.

### Thermo Gravimetric Analysis

Thermal decomposition analysis of scaffolds were analyzed using a Netzsch-Geratebau GmbH thermal analyzer TG (STA 409C) at a uniform scanning rate of 10 # C/min under the atmosphere of nitrogen. The differential thermogravimetric analysis (DTG) plot (%/min) indicates the maximum weight loss. Samples weighing around 5 mg each were subjected for thermal analysis.

### Differential Scanning Calorimetric (DSC) Analysis

Heat denaturation property of scaffolds was studied by Differential Scanning Calorimetry, Netzsch-Geratebau GmbH thermal analyzer with DSC 200 PC. Sample quantity of 5 mg each of scaffolds was subjected to analysis at uniform scanning rate of 2°C/min under the atmosphere of nitrogen.

### Tensile and Tear Tests

The automatic control electronic universal testing machine (UTM, H10KS, Tinius Olsen) according to the ASTM D 638-03 method was used to measure tensile parameters. Specimen length and diameter were measured using a reading microscope. The test was performed to determine the capability of a material to resist the deformation during stretched. Barrier properties (OTR) of the samples were characterized by Noselab Ats. The prepared films were determined on samples cutting into small pieces (2 cm × 3 cm). The samples were first dried in a vacuum drier at 60°C for 2 days. The WVTR of the Kc, Amn. and Amn-Kc films were calculated by Mocon Permatran, according to the standard of ASTM F 1249-90. Five samples were prepared and the average values were calculated. Specimens from the normal human amniotic membrane group were 25 mm long and 9.8–10.2 mm in diameter. Each specimen was preset by 10 repeated loading and unloading. The experimental temperature was close to normal human body temperature (36.5 ± testing machine, with a loading speed of 5 mm/min). To maintain humidity in the specimens, a liquid spray was continuously administered. Upon experimental completion, the following indices were automatically generated from the automatic control electronic UTM: maximum load, maximum displacement, maximum stress, maximum strain, elastic limit load, elastic limit stress, and stress-strain curve: 1.0°C.

### Water Uptake Capacity

Measured unit of dried scaffolds are weighed and placed in a watch glass filled with deionized water and retained for 20 min. Difference in the weight was measured before and after placing in watch glass filled with water to find out the actual water uptake capacity.

### Biomaterial Swelling Analyses

Biomaterial of 200 μm thickness were cut into 1 sq cm blocks and placed in petri dish with 10 ml PBS (pH 7.4) and incubated at RT for 1 week. After 1 week, biomaterials were photographed and the increase in thickness was measured.

### Fourier Transform Infrared Spectroscopy (FT-IR)

The chemical compatibility of prepared samples were analyzed by FT-IR spectroscopy. Infrared spectra (IR) of biomaterials was obtained using phase resolution 128, and averaging 25 scans/min, using a Bruker IFS 28 Equinox infrared spectrophotometer, equipped with an OPUS-2.52 software was used for data acquisition.

### Cell Viability Assay

To assess the effect of Amn, KC and Amn-KC on cell viability, the sheets were cut into circular patches to fit into a well of a 96-well plate. Fibroblast cells grown to 80% confluency were taken, trypsinized and plated into a 96-well plate at a density of 3000 cells per well. The plate was incubated overnight with DMEM, 10% FBS, Pen-Strep media, following incubated, the media was replaced with DMEM, 1X Pen-Strep with or without 10% FBS. Cut circular Amn, KC or Amn-KC membrane were presoaked in DMEM for 20 min and were added on top of the cells in 96-well plate. The plate was incubated for 2 days and cell viability was assessed using Cell Titer Glo kit according to the manufacturer’s protocol.

### Flow Cytometry Based Cell Cycle Analyses

To assess the effect of Amn, KC, and Amn-KC on cell cycle, human foreskin fibroblasts were grown in 10 cm petri plate till 80% confluency. Following which, the culture media was replaced with DMEM, 1X Pen-Strep only. Fifty milligram of Amn or KC, or Amn-KC was added to it and incubated for 2 days. Post incubation, the biomaterial was removed, cells were trypsinised, pelleted and fixed with 75% ethanol and stored at 4°C. Post fixation, cells were rehydrated with PBS following which propidium iodide (PI) staining was performed as described earlier (Andang et al., 2008). Flow cytometry was performed on FACScan instrument using CellQuest Pro software and the results were analyzed using FlowJo software (Tree Star, Ashland, OR, United States).

### Paraffin Sectioning

Human pre processed and post enzymatic (protease) processed placenta or Amn was embedded in paraffin as per standard procedure and 5 μm sections were collected on to a glass slide. For mice, on the 14th day, 0.5 cm^2^ of the healed skin was removed using excision method, fixed in 4% of PFA and embedded in paraffin as per standard procedure. Vertical sections (5 μm) were cut, and collected onto glass slides. The pathological changes were examined using microscopical examination.

### Tissue Histology

The sectioned slides containing either placenta, Amn or mice tissue were subjected to Haematoxylin and Eosin (HE) staining to look at the tissue architecture, Masson’s Trichrome (MT) staining to look at collagen content, Periodic acid-Schiff (PAS) stain to look at polysaccharide content, and Verhoeff’s stain to look at elastin content.

### Cell Extracts and Western Blotting

Cell extracts were performed by using RIPA buffer (Thermo Fisher Scientific). The samples were analyzed by western blotting using the following antibodies: anti-Collagen I and III (Abcam), anti-β actin (Millipore).

### PCR Array Analyses for Wound Healing Markers

To assess different markers participating in wound healing, Human Wound healing RT2 profiler PCR array (Qiagen) was utilized to assess 84 key genes that are central to wound healing. Human foreskin fibroblast cells were grown to 80% confluency and the culture media was replaced with DMEM and 1X Pen-Strep. To this set up, either 50 mg Amn, or 50 mg KC, 10 ng/ml EGF or 10 ng/ml FGF2 or 10 ng/ml TGFa was added and incubated for 2 days. Post incubation, cells were trypsinized, pelleted and total RNA was isolated using Trizol method as per manufacturer’s instruction. Total isolated RNA was then converted into cDNA using Superscript II (Invitrogen) and were taken for PCR array analyses as per manufacturer’s instructions. Fold change was calculated using delta CT method and was used to generate clustergrams using Euclidean distance metrics and average linkage method using the online analysis software provided by Qiagen^1^. Pairwise comparison of samples were done using Microsoft excel program.

### Zebrafish Developmental Toxicity Analyses

Fertilized zebrafish embryos at 1 cell stage were dechorionated and around 50 embryos were collected per petri dish in 30 ml egg water without methylene blue. To these plates, 50 mg of Amn or Kc or Amn-KC were added and embryos incubated for 2 days. Post 2 days incubation, the morphological features were observed and compared to untreated control embryos.

### Mouse Wounding and Biomaterial Application Studies

Healthy swiss albino mouse weighing about 25 g of either sex were selected for the present study, mouse were perfused and fixed individually under aseptic condition. Using the excisional wounding method, 0.5 mm^2^ of the epidermal, dermal, hypodermal and panniculus carnosus layers were removed completely (Dovi et al., 2003). Post wounding, animals were grouped into control without any biomaterial, treated group with Amn and treated group with Amn-KC. Biomaterials, Amn or Amn KC were cut according to the shape of the wound and placed onto the wound with the materials edges tucked underneath the surrounding skin. Since these materials were sterilized by UV and gamma irradiation eliminating the risk of infections of microbial origin, and were safe for *in vivo* wound heal applications. Animals were closely observed for any infection, and those which showed any sign of infection were separated, excluded from the study and replaced.

### Mouse Wound Healing Assessment

Assessment of wound healing was done by daily observation. Photographic documentation of wound was done to assess skin color, presence of wound exudates, dryness and wound closure. The reduction of wound diameter was calculated to reflect the progress of wound healing.

### Normal Flora Assessment

Wet swab method was used to collect floral sample from the healed wounds of the control and treatment animal groups. Samples were cultured using standard procedure and interpreted using microscopic examination. One swab per mouse was collected after carefully cleaning the wound with sterile water in order to prevent surface contamination. The samples were transported to the 1X PBS within 1 h of collection to prevent drying of the swabs. Swabs were immediately inoculated onto Blood agar, Nutrient agar, Tryptic Soy agar, and Salmonella-Shigella agar. MacConkey agar, Mannitol salt agar and Eosin methylene blue agar and incubated at 37°C aerobically for 24–48 h. Anaerobic cultures were not done due to logistic difficulties. Bacterial colonies on the agar plates were then Gram stained. These stained bacterial isolates were subjected to biochemical tests for further identification and classification. Bacterial pathogens were identified by conventional biochemical methods according to standard microbiological techniques. Most of the identified colonies were coagulase negative type of *Staphylococcus aureus*, and were uniform gram positive cocci.

## Results

### Screening and Hit Identification

**FIGURE 1:**
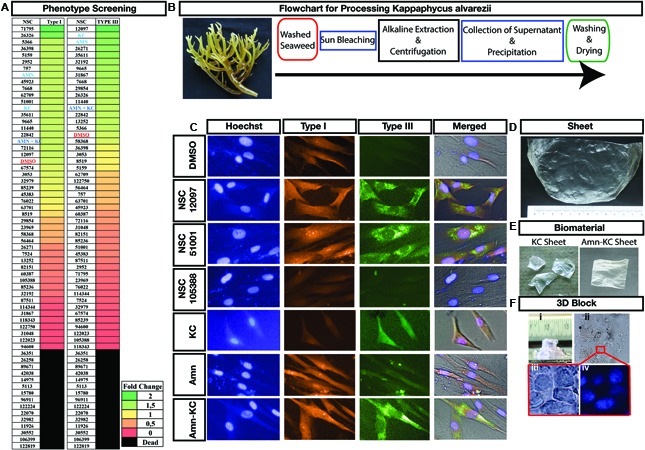
Phenotypic Screening and Material Development. **(A)** Phenotypic scoring of various compounds producing increase or decrease of type I or III collagen or lethal to fibroblast are shown as heatmap. Each compound is identified using their NSC number, except for Amnion, identified as Amn, *kappa carrageenan*, identified as KC and their combination, identified as Amn-KC. **(B)** Photograph of *Kappaphycus alvarezii* that was used as starting material for isolation of *kappa carrageenan* and Flowchart showing the processing of *K. alvarezii* to isolate KC in order to make the biomaterial. **(C)** Representative images of fibroblast showing collagen Type I (red) or III (green) staining post treatment with compounds shown with their NSC numbers, Amn, KC, Amn-KC when compared to DMSO. The nuclei of the cells are stained with Hoechst (in blue) and all the image panels are overlaid on a bright field image under merged. **(D)** Biomaterial KC post development and sterilization. **(E)** Pieces of KC and Amn-KC post sterilization. **(F)** Biomaterial KC generated 3D structure (i) with cells cultured inside (ii) zoomed images of cells are shown in brightfield (iii) and DAPI (blue) staining (iv).

A simple phenotype based screen quantifying types I and III collagen was conducted on human fibroblast cells. The screen allowed simultaneous assessment of both the types of collagen in the same cells. Collagen types I and III are the major components of the skin (Lovell et al., 1987) and during wound healing, it is typically produced by the fibroblast cells moving into the wound site (Rittie, 2016). Post screening and quantification, the effect of compounds could be grouped into three categories, category 1 produced no visible change when compared to control cells. A total of 47.5% of the screened compounds belonged to this category. A total of 12.5% of all screened compounds producing lethality belonged to category 2 (**Figure 1A**). Category 3 consists of 40% of screened compounds that produced an effect on collagen production (**Figure 1A**). Among category 3 compounds, a total of 20 and 16 compounds increased the production of types I and III collagen, respectively. A total of 28 and 32 compounds decreased the production of types I and III collagen, respectively. Out of 20 compounds, 13 increased type I collagen and had no or decreased effect on type III. Similarly, out of 16 compounds, 9 increased type III with no or decreased effect on type I. A total of 5 compounds increased both the types of collagen tested here (**Figure 1A**). Carefully examining the compounds increasing secretion of both the types, we identified sulphated polysaccharide *kappa carrageenan* (KC) derived from seaweed *K. alvarezii.* Compound KC is a commonly used food additive that also has well established anti-inflammatory (de Brito et al., 2013), anti-cancer, antimicrobial, and wound healing properties (Aguilar-Briseno et al., 2015; Hadj Ammar et al., 2015). Therefore, KC could be a good component that can be combined with amnion to modulate local wound environment. Developing a biomaterial by incorporating KC with Amnion would help in decreasing inflammation at wound site and absorb exudates. The excess proteases in the wound site could degrade collagen from amnion instead and the antimicrobial activity of sulphated polysaccharide KC would decrease microbial load thereby removing impediments of wound healing.

### Biomaterial Preparation

We focused on harnessing the properties of KC into a biomaterial so as to modulate local wound environment and aid in healing. As a first step we generated two biomaterials, one from KC alone and the other was a combination of KC and amnion to generate Amn-KC (**Figures 1B,D,E**) and characterized them in comparison to amnion material (Amn). A protocol was developed to extract sulfated polysaccharide, *kappa-carrageenan* from seaweeds to make 200 μm thick scaffold (KC) (**Figures 1B,E**) and mixing of Amn and KC generated 200 μm thick Amn-KC scaffold (**Figure 1E**). A straight forward phenotypic screening used here allowed for visualization of types I and III in fibroblast cells (**Figure 1C**). When compared to DMSO, few compounds such as NSC12097 and NSC51001 clearly showed an increase in both types while NSC105388 displayed a decrease of both the types of collagen (**Figure 1C**). In comparison, KC produced an increase in Type III while Amn clearly produced an increase in Type I (**Figure 1C**). Combining Amn and KC increased the production of both the collagen types when compared to DMSO treated cells. These results indicated that a combination of both Amn and KC could indeed enhance production of both the types of collagen.

### Characterization of the Biophysical Properties of These Biomaterials

**FIGURE 2:**
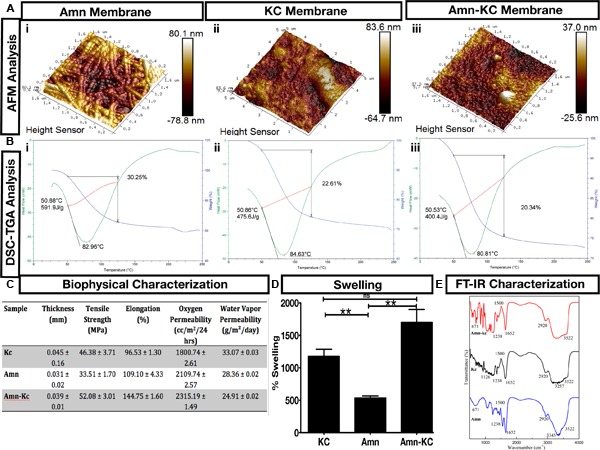
Biomaterial Characterization. **(A)** Atomic force microscopy (AFM) and **(B)** differential scanning calorimetry (DSC) – thermogravimetry (TGA) measurements of (i) Amn, (ii) KC, and (iii) Amn-KC. **(C)** Biophysical characterization measuring thickness, tensile strength, elongation, oxygen permeability and water vapor permeability of Amn, KC, and Amn-KC. **(D)** Biomaterial swelling capacity shown in percentage for Amn, KC, and Amn-KC. **(E)** Fourier transform infrared spectroscopy (FT-IR) characterization of Amn, KC, and Amn-KC with different peak values shown for each membrane. Data represent mean ± SD, ^∗∗^P < 0.01 compared to their initial thickness before swelling.

Biomaterial KC not only allowed generation of large sheets (**Figures 1D,E**), but also 3D scaffolds (**Figure 1F**) and was conducive for culturing of cells inside 3D scaffolds (**Figure 1F**). This demonstrated a molding capacity and suitability of these biomaterials for growing cells. Biophysical characterization was done to understand the nature of these materials. Analysis using AFM showed columns of collagen bundles arranged in a mesh like structure in Amn (**Figure 2A**). Comparing Amn material to KC and Amn-KC clearly showed that KC and Amn-KC were having a smooth topology when compared to Amn (**Figure 2A**). Thermal analysis using differential scanning calorimetry-thermogravimetry (DSC-TGA) showed that the materials were relatively stable at wide range of temperature and did not display any alteration in their composition suggesting the preservation of collagen triple helical structures as well as interaction of various constituents to stabilize the material (**Figure 2B**). Materials also showed a strong tensile strength, oxygen and water vapor permeation and elongation capacity (**Figure 2C**). Material swelling experiments showed that out of the three materials analyzed, Amn-Kc had the highest swelling capacity when compared to Amn and KC (**Figure 2D**). The chemical compatibility of prepared samples were analyzed by FT-IR spectroscopy (**Figure 2E**). The biomaterial Amn showed all necessary peaks of collagen in IR bands at the region of 1646 cm^-1^ (C=O stretching) for amide I; 1550 cm^-1^ (N–H bending) for amide II; 1247 cm^-1^ (C–N stretching) for amide III, and 3428 cm^-1^ for O–H stretching. The material KC showed various distinct peaks in the region of very broad band spreading (3150–3600 cm^-1^) due to polyhydroxy OH group; 2955 cm^-1^due to C-H stretch; 1268 cm^-1^ due to S=O of sulfate esters; 1078 cm^-1^ due to C=O stretch of cyclic ethers; 926 cm^-1^ due to C=O stretch of polyhydroxy groups attached to carbons; 848 cm^-1^ due to C–O–S of axial sulfate on C-4 of galactose (**Figure 2E**). The FTIR spectra of prepared Amn-KC composite shows all characteristic peaks of Amn and KC, which implies good integration of Amn and Kc (**Figure 2E**). Hence, it is anticipated that the composite material may very well inherit the pharmacological activities of the individual constituent *viz*., Amn and KC. The biophysical characterization displayed that the material was not toxic to cells and could act as a scaffold to stabilize wound structure while keeping it aerated and moist. In addition, the material could gradually imbibe exudates and dead debris from wound site.

### Characterization of *In Vitro* Effects of the Biomaterials

**FIGURE 3:**
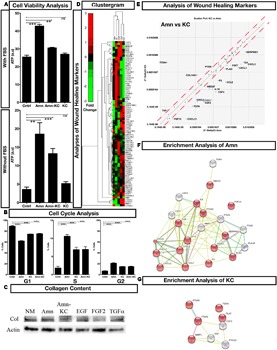
Functional Profiling of Biomaterial. **(A)** Cell viability analysis of fibroblast cells grown with Amn, KC, and Amn-KC in the presence or absence of fetal bovine serum (FBS). **(B)** Flow cytometry based cell cycle analysis of cells when grown with Amn, KC, and Amn-KC. **(C)** Western blot analysis of total collagen content of cells grown with Amn and Amn-KC when compared to growth factors EFG, FGF2 or TGFα. **(D)** Clustergram generated from PCR array analysis of all wound healing markers with fold change represented as color codes. **(E)** Differential expression of wound healing markers when cells are grown on Amn or KC. **(F,G)** Gene enrichment analysis showing unique subset of genes stimulated post treatment with Amn **(F)** or KC **(G)**. Data represent mean ± SD, ^∗∗^P < 0.01, ^∗∗∗^P < 0.001 compared to control.

The *in vitro* effects were understood by growing human foreskin fibroblast cells on biomaterials. Fibroblasts cultured with Amn displayed increased cell viability when compared to Amn-KC or KC, even after serum was withdrawn from the medium (**Figure 3A**). These results indicate that the biomaterials were actively promoting cell viability. To examine whether this increase in viability was due to increase in proliferation of cells, FACS based cell cycle analyses was performed to quantify cells in different phases of cell cycle. In the absence of serum, control cells grown without biomaterial displayed arrest in G1 phase of cell cycle; however, the cells grown with biomaterials showed an increase of cells in S and G2 phases indicating that these materials promoted cell cycle thereby proliferation of cells even in the absence of serum (**Figure 3B**). These results show that the biomaterials promote proliferation and viability of fibroblast cells. Given that fibroblast cells are very important players in the process of wound healing (Wong et al., 2007), materials that promote its viability and increase its proliferation will have an advantageous effect on increasing the healing efficiency. Various growth factors such as epidermal growth factor (EGF), fibroblast growth factor (FGF), and transforming growth factor (TGF) have been extensively used to increase fibroblast cell proliferation and to promote chronic wound healing with varying degree of success (Grazul-Bilska et al., 2003). Growth factor therapy has encountered various roadblocks such as decreased site availability due to higher protease activity at wound site, scarring and activation of other signaling cascades, therapy cost and duration (Grazul-Bilska et al., 2003). In addition, these growth factors have also been linked to epithelial malignancies making them less attractive for long-term therapeutic application (Aaronson et al., 1990). These biomaterials with their beneficial effect on cell viability and proliferation of fibroblast cells, so, offer a very good alternative to growth factor based therapy without having the side effects. To examine the effect of biomaterial on total cellular collagen I and III contents, western blot analyses was done on cells grown in the presence of Amn and Amn-KC and compared to cells grown in the presence of EGF, FGF2, and TGFα. The results showed that similar to growth factor treatment, biomaterials Amn and Amn-KC allowed cells to produce collagen (**Figure 3C**). Since collagen types I and III constitute a major portion of fibrous structural proteins in the pre-and post-healed skin, these results indicate that these biomaterials not only promote proliferation and viability of fibroblast cells, but also allow collagen secretion.

To assess the effect of these biomaterials on various steps of wound healing, we performed expression profiling of 84 different key wound healing markers necessary for inflammation, granulation and tissue remodeling and compared it to the effect produced by growth factors EGF, FGF2, and TGFα. Clustering based on expression of the markers identified that treatment with biomaterial produced expression profile that was similar to that of EGF treatment (**Figure 3D**, **Supplementary Figure S1**, and **Table S1**) with an enrichment for positive regulator of cell migration seen in Amn treatment and regulators essential for wound healing in KC treatment (**Figures 3E–G**, **Supplementary Figure S1**, and **Table S1**). Comparing Amn and KC, no dramatic change in the expression of most of the markers was seen; however, each material had a subset of markers that was unique to it. Treatment with Amn elevated expression of positive regulators for cell migration (**Figure 3E**). Expression of various cytokines and chemokines such as CXCL1, 2, 5, 11, CCL2, 7, IL1B, IL6, growth factors such as FGF7, 10, TGFA, CSF2, 3, HBEGF, and remodeling enzymes such as F3, PLAU, SERPINE1 which are known regulators of cell migration in wound healing (Werner and Grose, 2003) were enriched in Amn treatment when compared to KC (**Figures 3D,E**, **Supplementary Figure S1**, and **Table S1**). Treatment with KC saw increase of ITGA4 in particular, when compared to Amn (**Figure 3D**). Integrin alpha chain family of proteins to which ITGA4 belongs to, play an important role in cellular adhesion and cell-cell/cell-ECM interaction in wound healing (Hynes et al., 2002; DiPersio et al., 2016). These results demonstrate that application of biomaterials trigger the expression of markers essential for wound healing. Both Amn and KC enhanced unique subsets of markers required for cell migration and wound healing, respectively.

### *In Vivo* Effects of the Biomaterials

**FIGURE 4:**
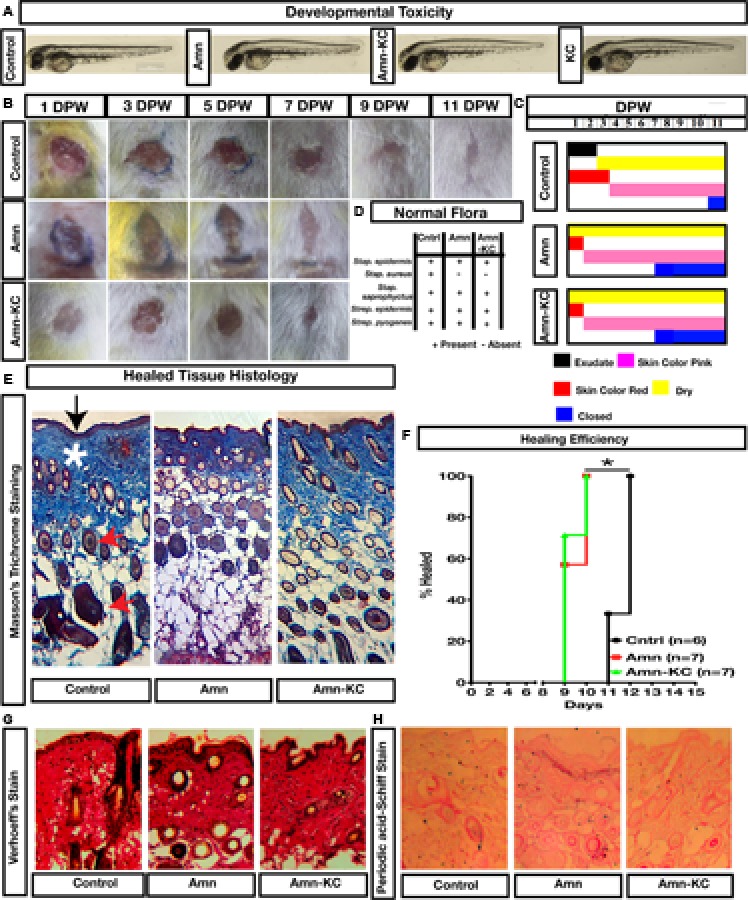
Preclinical evaluation of biomaterial. **(A)** Zebrafish based evaluation of developmental toxicity. One-cell Zebrafish embryos exposed to Amn, KC or Amn-KC were photographed at 2 days post fertilization (dpf). **(B)** Mouse model based wound healing evaluation of Amn or Amn-KC material, images of days post wounding (DPW) showing the efficiency of healing. **(C)** Physical observation of the wound at different time point post treatment. **(D)** Mouse normal flora of skin analyses post healing of wound after treatment with Amn, Amn-KC when compared to untreated mouse. **(E)** Masson’s trichrome staining of mice skin post healing showing collagen content in deep blue color (shown in asterix) and epidermis (shown in arrow). **(F)** Kaplan Meier plot showing percent of mouse healed in different treatments. **(G)** Verhoeff’s staining showing elastin content of healed tissue. **(H)** Periodic acid-Schiff staining showing polysaccharide content of healed tissue.

The biophysical analysis and *in vitro* analyses pointed out that although Amn and KC have a capacity to promote healing, their combination might synergistically promote their performance. Since application of Amn to wounds has been demonstrated to be efficient, we chose to perform *in vivo* evaluation of Amn-KC and compared it to Amn. Zebrafish and mouse models were used to assess the *in vivo* effects of these materials. The materials did not produce any developmental defects in zebrafish model indicating that they did not interfere with the molecular cascade governing early zebrafish development (**Figure 4A**). Preclinical evaluation of wound healing potential of Amn and Amn-KC was done using mice. One day post-wounded (DPW) mice were grouped into untreated control or treated with Amn or Amn-KC material (**Figure 4B**). As the biomaterials were digested and absorbed at the wounded site, fresh biomaterial was replaced every 2 days. The results showed that when compared to controls, Amn and Amn-KC mice did not have wound exudates and the skin color changed from red to healthy pink already by 2 DPW (**Figures 4B,C**). More than 50% of the Amn and Amn-Kc treated mice showed wound closure by 9 and 7 DPW, respectively, and all of them had closed wounds by 12 and 9 DPW (**Figures 4B,C,F**) respectively. Control mice wounds, on the other hand were characterized by exudates on 1 DPW and the wounded skin turned pink from 4 DPW (**Figure 4B,C**). Signs of wound closure were seen from 10 DPW and all of them had healed completely by 15 DPW (**Figure 4B,C**). Normal flora of the skin was done to assess the effect of biomaterial treatment on re-colonization of most common bacterial strains on mice skin. The analysis showed that except for the absence of *S. aureus* on the skin of the Amn and Amn-Kc mice, the rest of the *Staphylococcus* and *Streptococcus* were detected in all the groups (**Figure 4D**). Tissue histology of the healed skin tissue by various staining methods displayed a healthy epidermis with epithelial layer, connective tissue substances such as collagen, elastin, proteoglycans, hyaluronic acid and numerous hair follicles in all the groups (**Figures 4E,G,H**). These results indicate that the observed physical properties of biomaterials can stabilize wound structure, act as an absorbent gradually removing exudate, proteases and other secretary inflammatory factors, reduce microbial growth, avert drying, allow oxygen and water vapor permeation, is absorbed at wound site, promotes regeneration of healthy skin tissue, healing and wound closure. In addition, they facilitate fibroblast growth, collagen production and stimulate expression of markers that promote wound healing.

## Discussion

Wound management places an enormous burden on healthcare system and has to be dealt by controlling both local wound environment and systemic effects (Harding et al., 2002). Wound healing is a multifaceted skin regenerative process comprising the regulatory events of suppression of inflammatory reactions and opportunistic infections, induction of proliferation of connective tissue cells such as fibroblasts, collagen deposition and remodeling of ECM (Martin, 1997). Effective wound therapeutic biomaterials should induce conversion of cells at the wound site from senescence to active state so as to perform the above functions for achieving the complete regeneration of healthy skin. Bioactive molecules isolated from natural products offer a good addition to existing biomaterials in order to enhance their performance. Employing a simple phenotype based screen, described here could form a platform to identify and formulate natural product based biomaterial development. The ease for preparing these materials offers a cheaper alternative to existing therapies and their biophysical characterization indicates that the material permits modulating the local wound environment and by that its systemic effects. Tensile strength of the materials developed here reinforces the desirable mechanical properties suitable for handling them for clinical applications, while permeability to oxygen and water vapor allow the wound to prevent hypoxia and desiccation as well as to facilitate diffusion of wound heal substances, the important factors in wound healing (Hunt and Pai, 1972; Collawn et al., 1998; Junker et al., 2013a,b; Chaudhari et al., 2016). Thin width, rough topology, and transparent nature allow easy application of the material and visual monitoring of wound site. Hydrodynamic properties such as capacity to swell, due to the addition of carrageenan sulphated polysaccharides, allows the material to expand by absorbing wound exudate together with impediments such as excess proteases and inflammatory agents while keeping the wound moist and favoring repair by facilitating cell attachment, proliferation and migration (Payam et al., 2010). These results demonstrate that these porous hydrophilic biomaterials offer an affordable, practical and an uncomplicated way for modulating wound environment.

Collagen or collagen rich amnion has a demonstrated applicability for wound healing (Ilic et al., 2016; Guo et al., 2017) and is a widely accepted as a safe and multifunctional material can also serve as substrate for the excess proteases from the wound site, thereby decreasing protease-mediated impediment to wound healing (Chen et al., 1999; Suzuki et al., 2000; Matthews et al., 2002; Chan et al., 2007). The marine sourced sulfated polysaccharides have established anti-oxidative, antimicrobial, anticoagulant, and anticancer activities (Freudenberg et al., 2015; Song et al., 2015; Fernando et al., 2016; Prasedya et al., 2016; Wang et al., 2017). Biomaterial developed here capitalizes on the favorable properties of these individual components to regulate microenvironment thereby acting synergistically to promote healing. Natural polymers present in Amn-KC biomaterials such as hyaluronic acid and carrageenan are negatively charged mainly because of the presence of sulfate groups along the chain. Though the influence of the charge type and density on cellular response is not completely understood, negatively charged biomaterials are known to suppress inflammatory response in contrast to positively charged polymers that tend to attract inflammatory cells (Bhatia et al., 2005).

The biomaterials used here not only stimulate fibroblast proliferation and collagen secretion, they also regulate expression of several genes positively associated with wound healing. Among all the genes enriched with Amn treatment, FGF7 has a specific mitogenic activity for epithelial cells, more specifically in keratinocytes (Werner and Grose, 2003). An increase in its expression is seen in fibroblast post injury, and previous reports suggest that FGF7 induces hyper proliferation of keratinocytes and enhanced wound closure (Erdag et al., 2004). Increase in expression of FGF7 is found in N-WASP conditional knockout mice which shows hyper proliferation of keratinocytes and rapid wound closure via TGFβ signaling mechanism (Jain et al., 2016). Expression of colony stimulating factors, CSF3 in particular, is also increased post Amn treatment. CSF3 is very crucial for keratinocyte-fibroblast cross talk (Carr et al., 2017). CSF3 is an important hematopoietic growth factor that has been shown to increase proliferation of keratinocytes and is also undergoing phase IV clinical trial for burn wounds (Nakazawa et al., 2002; Liu et al., 2016). Treatment with KC saw an increased expression of ITGA4 when compared to Amn. Integrin ITGA4 has been shown to have important physiological roles, especially in regulating immune system function, such as homing ability of T-cells (Yang et al., 1995, 1996; Arroyo et al., 1996, 1999). ITGA4 has been shown for its binding to ligands such as family of ADAM proteins (Eto et al., 2002), fibronectin (Liao et al., 2002), EMILIN1 (Danussi et al., 2011), thrombospondin-1 (Staniszewska et al., 2007), VCAM-1 (Takahashi et al., 2000), and all these ligands play an essential role in mediating signal transduction important for cell adhesion, migration, angiogenesis and dermal fibroblast and keratinocyte proliferation. Many of these genes serve as therapeutic targets, and various clinical trials are being conducted using recombinant proteins of these genes to evaluate their potential for wound healing. These materials overcome the need to apply recombinant proteins and offer an affordable and efficient way to modulate the expression of these genes at wound site. Most importantly, it is observed that Amn-KC down regulates the expression of chemokines such as IL1 and IL6 and the effect is more pronounced when compared to treatment with individual components, suggesting the anti-inflammatory function of the biomaterials. It is noteworthy that the biomaterial shows increase and decrease in the expression levels of wound heal biomarkers such as tissue inhibitor matrix metalloproteinase (TIMP) and matrix metalloproteinases (MMPs) respectively. Though we are not able to give the complete expression profiling of MMPs and TIMPs due to complex cellular interaction with the biomaterials, we can find that there is an up regulation of TIMP1 and down regulation of MMP1 indicating the environment favorable for collagen secretion and tissue remodeling. It is also to be noted that there are variations in the expression of these proteins when Amn and Kc separately treated along with other growth factors. Wound healing is marked by the dynamic secretion of collagen proteins by fibroblasts. In acute wounds, the secretion of MMPs during the early phase followed by secretion of MMP inhibitors (TIMPs) to facilitate skin regeneration. However, the MMP levels are abnormally elevated in the chronic wound with down regulation of expression of TIMPs (Fleck and Simman, 2010). The elevated ratio of MMPs to TIMPs leads to excessive ECM degradation as several MMPs have collagenolytic properties. The Amn-KC has the tissue regenerative potential as it can not only induce the expression of TIMPs but also down regulate MMPs, suggesting their potential for healing the chronic wounds. It is reported that several marine natural products including sulfated polysaccharides have the ability to inhibit MMPs (Zhang and Kim, 2009). However, reports on modulation of expression of MMPs and TIMPs are scarce for KC and a report indicates the potential of KC to down regulate MMP2 (Chen et al., 2007). KC and Amn constituents can inhibit MMPs, induce TIMPs or regulate the expression of them to favor healing (Zhang and Kim, 2009). The observations of the study substantially suggest that the Amn-Kc material has protective and proliferative effects on fibroblasts in addition to the chemotactic effects of collagen and carrageenan on cell adhesion, cell differentiation, and tissue regeneration.

The physico-chemical properties of the materials developed here, their effect *in vitro* and *in vivo*, and their pre-clinical evaluation indicate that biomaterial based gradual alteration to wound site is a promising and affordable approach to heal wounds. This gradual alteration to wound site is more desirable to direct intervention therapies, such as use of growth factors, as it provides an opportunity for individual’s physiology to slowly adapt to the changes to the wound site. In addition, these biomaterials avoid the challenges and malignancies that are often associated with growth factor and other interventional therapies.

## Conclusion

In this report, we demonstrate that by phenotype screen we could identify bioactive natural products whose properties could be harnessed for developing biomaterials with favorable physico-chemical characteristics. These biomaterials can gradually alter wound site and synergistically promote healing. Promoting healing via systemic effects produced through modulating local wound environment might be an appropriate means to engage individual’s physiology to participate in healing. Removal of impediments produces systemic effects by decreasing inflammation, allows fibroblast to proliferate and secrete collagen to repair wound. The uncomplicated nature of this approach and higher degree of healing of wounds by means of anti-inflammatory potential as well as creating an environment favorable for healing strongly advocate its application in therapy for a range of acute and chronic wounds, pressure and venous ulcers, burns injury, etc. In addition, they offer a cheaper alternative to existing therapy such as use of growth factors, which have been widely associated with various epithelial malignancies (Janmaat and Giaccone, 2003).

## Ethics Statement

All animal works were performed in accordance with the national guidelines and local ethical committee constituted at Loyola College, Chennai, CSIR-Central Leather Research Institute, Chennai or Karolinska Institutet, Sweden. The study protocols were verified and approved by the ethical committee. The use of zebrafish and the study has been approved by the Stockholm North Animal Committee, Dnr N122/15. Rodent study was approved by ethical committee number IAEC/ERI/LC/03/17 and IAEC NO 03/2015(A).

## Author Contributions

SK designed the work; SS, GC, SSA, RT, MS, RK, and HH performed experiments; SV, BM, PG, and SK provided material support. All authors participated in data analysis and writing.

## Conflict of Interest Statement

The authors declare that the research was conducted in the absence of any commercial or financial relationships that could be construed as a potential conflict of interest.

## Footnotes

1 http://www.qiagen.com/fi/shop/genes-and-pathways/data-analysis-center-overview-page/

## Supplementary Material

FIGURE S1 PCR array plots for various wound healing markers. PCR array based scatter plots comparing the effects of Amn or KC treated fibroblast cells with EGF, FGF2, and TGFa.

TABLE S1 Table showing up and down regulated genes identified through PCR Wound Healing Array. Genes that are up or down regulated in Amn or KC or in comparison to EGF, FGF2, and TGFa growth factors identified through PCR array for wound healing analysis.
