# Supplementary figures and images for: Phenotypic Screening Identifies Synergistically Acting Natural Product Enhancing the Performance of Biomaterial Based Wound Healing
Source: Front Pharmacol. 2017 Jul 18;8:433. doi: 10.3389/fphar.2017.00433 (PMC5513901; doi:10.3389/fphar.2017.00433)

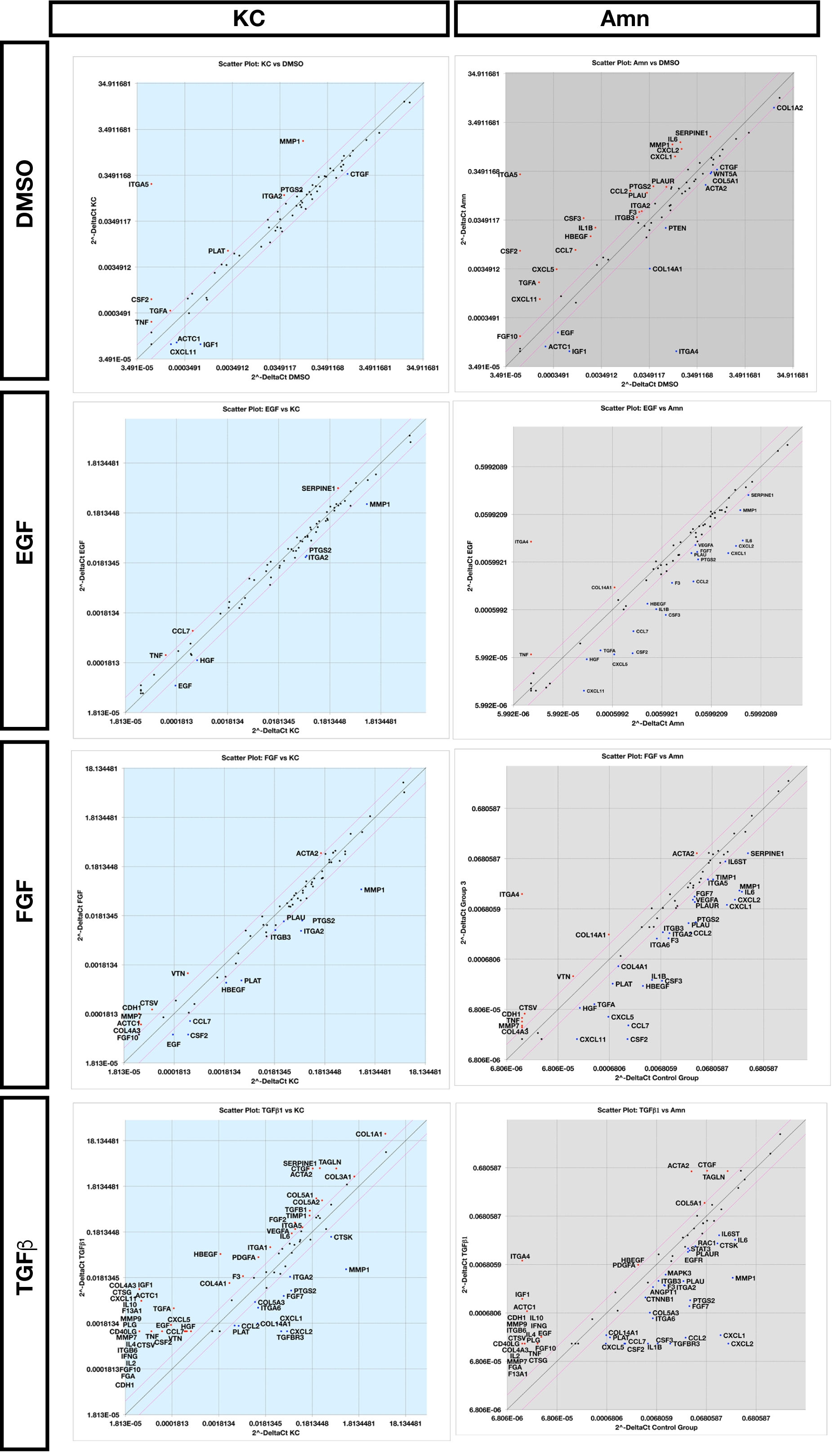

Supplement: FIGURE S1 — PCR array plots for various wound healing markers. PCR array based scatter plots comparing the effects of Amn or KC treated fibroblast cells with EGF, FGF2, and TGFa. [file Image_1.TIF]
