# Supplementary material for: Phenotypic Screening Identifies Synergistically Acting Natural Product Enhancing the Performance of Biomaterial Based Wound Healing
Source: Front Pharmacol. 2017 Jul 18;8:433. doi: 10.3389/fphar.2017.00433 (PMC5513901; doi:10.3389/fphar.2017.00433)
Supplement: TABLE S1 — Table showing up and down regulated genes identified through PCR Wound Healing Array. Genes that are up or down regulated in Amn or KC or in comparison to EGF, FGF2, and TGFa growth factors identified through PCR array for wound healing analysis. [file Table_1.PDF]

Upregulated								
Amn	KC	KC vs Amn	EGF vs Amn	EGF vs KC	FGF2 vs Amn	FGF2 vs KC	TGFβ1 vs Amn	TGFβ1 vs KC
CCL2	CSF2	COL14A1	COL14A1	CCL7	ACTA2	ACTA2	ACTA2	ACTA2
CCL7	ITGA2	ITGA4	ITGA4	SERPINE1	CDH1	ACTC1	ACTC1	ACTC1
CSF2	ITGA5	PTEN	TNF	TNF	COL14A1	CDH1	CD40LG	CCL7
CSF3	MMP1	TNF			COL4A3	COL4A3	CDH1	CD40LG
CXCL1	PLAT				CTSV	CTSV	COL4A3	CDH1
CXCL11	PTGS2				ITGA4	FGF10	COL5A1	COL1A1
CXCL2	TGFA				MMP7	MMP7	CTGF	COL3A1
CXCL5	TNF				TNF	VTN	CTSG	COL4A1
F3					VTN		CTSV	COL4A3
FGF10							EGF	COL5A1
HBEGF							F13A1	COL5A2
IL1B							FGA	CSF2
IL6							FGF10	CTGF
ITGA2							HBEGF	CTSG
ITGA5							IFNG	CTSV
ITGB3							IGF1	CXCL11
MMP1							IL10	CXCL5
PLAU							IL2	EGF
PLAUR							IL4	F13A1
PTGS2							ITGA4	F3
SERPINE1							ITGB6	FGA
TGFA							MMP7	FGF10
							MMP9	FGF2
							PDGFA	HBEGF
							PLG	HGF
							TAGLN	IFNG
							TNF	IGF1
								IL10
								IL2
								IL4
								IL6
								ITGA1
								ITGA5
								ITGB6
								MMP7
								MMP9
								PDGFA
								PLG
								SERPINE1
								TAGLN
								TGFA
								TGFB1
								TIMP1
								TNF
								VEGFA
								VTN

Downregulated								
Amn	KC	KC vs Amn	EGF vs Amn	EGF vs KC	FGF2 vs Amn	FGF2 vs KC	TGFβ1 vs Am	TGFβ1 vs KC
ACTA2	ACTC1	CCL2	CCL2	EGF	CCL2	CCL7	ANGPT1	CCL2
ACTC1	CTGF	CCL7	CCL7	HGF	CCL7	CSF2	CCL2	COL14A1
COL14A1	CXCL11	CSF2	CSF2	ITGA2	COL4A1	EGF	CCL7	COL5A3
COL1A2	IGF1	CSF3	CSF3	MMP1	CSF2	HBEGF	COL14A1	CTSK
COL5A1		CXCL1	CXCL1	PTGS2	CSF3	ITGA2	COL5A3	CXCL1
CTGF		CXCL11	CXCL11		CXCL1	ITGB3	CSF2	CXCL2
EGF		CXCL2	CXCL2		CXCL11	MMP1	CSF3	FGF7
IGF1		CXCL5	CXCL5		CXCL2	PLAT	CTNNB1	ITGA2
ITGA4		F3	F3		CXCL5	PLAU	CTSK	ITGA6
PTEN		FGF10	FGF7		F3	PTGS2	CXCL1	MMP1
WNT5A		FGF7	HBEGF		FGF7		CXCL2	PLAT
		HBEGF	HGF		HBEGF		CXCL5	PTGS2
		IL1B	IL1B		HGF		EGFR	TGFBR3
		IL6	IL6		IL1B		F3	
		PLAU	MMP1		IL6		FGF7	
		SERPINE1	PLAU		IL6ST		IL1B	
		TGFA	PTGS2		ITGA2		IL6	
			SERPINE1		ITGA5		IL6ST	
			TGFA		ITGA6		ITGA2	
			VEGFA		ITGB3		ITGA6	
					MMP1		ITGB3	
					PLAT		MAPK3	
					PLAU		MMP1	
					PLAUR		PLAT	
					PTGS2		PLAU	
					SERPINE1		PLAUR	
					TGFA		PTGS2	
					TIMP1		RAC1	
					VEGFA		STAT3	
							TGFBR3	
